# Associations of CBC-Derived inflammatory indicators with sarcopenia and mortality in adults: evidence from Nhanes 1999 ∼ 2006

**DOI:** 10.1186/s12877-024-05012-2

**Published:** 2024-05-16

**Authors:** Botang Guo, Xinqing Liu, Qi Si, Dongdong Zhang, Minyao Li, Xi Li, Yang Zhao, Fulan Hu, Ming Zhang, Yu Liu, Dongsheng Hu

**Affiliations:** 1 Department of General Practice, The Affiliated Luohu Hospital of Shenzhen University Medical School, YouYi Road 47, Shenzhen, 518000 Guangdong People’s Republic of China; 2 College of Medical Administration, Harbin Medical University, Heilongjiang Province, Harbin, 150078 China; 3 Department of Epidemiology and Health Statistics, College of Public Health, Zhengzhou University, Henan Province, Zhengzhou, 450001 China; 4 Department of Biostatistics and Epidemiology, School of Public Health, Shenzhen University Medical School, Shenzhen, 518073 Guangdong Province China

**Keywords:** CBC-derived inflammatory indicators, Sarcopenia, Mortality, Random survival forests, NHANES

## Abstract

**Background:**

It has been proposed that inflammation plays a role in the development of sarcopenia. This study aimed to investigate the links of complete blood cell count (CBC) parameters and CBC-derived inflammatory indicators with sarcopenia and mortality.

**Methods:**

Data pertaining to sarcopenia were extracted from the 1999–2006 National Health and Nutrition Examination Survey (NHANES), and mortality events were ascertained through the National Death Index up to December 31, 2019. The CBC-derived inflammatory indicators assessed in this study included the neutrophil-to-lymphocyte ratio (NLR), derived neutrophil-to-lymphocyte ratio (dNLR), monocyte-to-lymphocyte ratio (MLR), neutrophil-monocyte to lymphocyte ratio (NMLR), systemic inflammatory response index (SIRI), and systemic immune-inflammation index (SII). The prognostic significance of these CBC-derived inflammatory indicators was evaluated using the random survival forests (RSF) analysis.

**Results:**

The study encompassed a cohort of 12,689 individuals, among whom 1,725 were diagnosed with sarcopenia. Among individuals with sarcopenia, 782 experienced all-cause mortality, and 195 succumbed to cardiovascular causes. Following adjustment for confounding variables, it was observed that elevated levels of NLR, dNLR, NMLR, SIRI, and SII were associated with an increased prevalence of sarcopenia. Among participants with sarcopenia, those in the highest quartile of NLR (HR = 1.336 [1.095–1.631]), dNLR (HR = 1.274 [1.046–1.550]), MLR (HR = 1.619 [1.290–2.032]), NMLR (HR = 1.390 [1.132–1.707]), and SIRI (HR = 1.501 [1.210–1.862]) exhibited an elevated risk of all-cause mortality compared to those in the lowest quartile of these inflammation-derived indicators. These associations were similarly observed in cardiovascular mortality (HR = 1.874 [1.169–3.003] for MLR, HR = 1.838 [1.175–2.878] for SIRI). The RSF analysis indicated that MLR exhibited the highest predictive power for both all-cause and cardiovascular mortality among individuals with sarcopenia.

**Conclusions:**

Our findings underscore the association between CBC-derived inflammatory indicators and mortality in adults with sarcopenia. Of note, MLR emerged as the most robust predictor of all-cause and cardiovascular mortality in this population.

**Supplementary Information:**

The online version contains supplementary material available at 10.1186/s12877-024-05012-2.

## Introduction

Sarcopenia, characterized by age-related muscle mass and strength decline, represents a significant health concern, particularly among the elderly population [S1, S2]. Its prevalence ranges from 5 to 13% in individuals aged 60 to 70 years, escalating to as high as 50% among those aged over 80 years [1]. Furthermore, sarcopenia imparts a spectrum of physical and functional limitations, exerting a profound impact on an individual’s quality of life and the ability to perform routine activities [2, 3]. This condition is associated with heightened vulnerability to physical disability, falls, fractures, and an elevated risk of mortality [4]. The etiology of sarcopenia is multifaceted, encompassing genetic, environmental, and lifestyle factors [5, 6]. Recent investigations, however, have revealed an intricate interplay between sarcopenia and the immune system, with inflammation emerging as a potential pivotal contributor to the onset of sarcopenia [7]. Consequently, further exploration of the nexus between sarcopenia and inflammation-related indicators holds promise for advancing our understanding and the development of novel interventions for this condition.

Inflammation constitutes a natural immune response triggered by potential threats, and it can underlie the development and progression of various chronic ailments [S3, S4]. Among older adults, inflammation has been implicated in the pathogenesis and progression of several maladies, encompassing cardiovascular diseases, respiratory conditions, and malignancies [8, 9]. The complete blood count (CBC) serves as a routine laboratory assessment that enumerates various blood constituents, including white blood cells, red blood cells, and platelets [10]. Notably, certain CBC-derived inflammatory indicators, such as the neutrophil-to-lymphocyte ratio (NLR), derived neutrophil-to-lymphocyte ratio (dNLR), monocyte-to-lymphocyte ratio (MLR), neutrophil-monocyte to lymphocyte ratio (NMLR), systemic inflammatory response index (SIRI), and systemic immune-inflammation index (SII), hold significance in the diagnosis and management of a spectrum of ailments [11–13]. For instance, these indicators have been linked to elevated mortality risk in adults afflicted with asthma [14]. In individuals with non-small-cell lung cancer (NSCLC), high levels of MLR and SII prior to surgery have demonstrated substantial associations with postoperative survival [15].

Inflammatory markers derived from CBC analyses have emerged as a promising avenue for identifying individuals at heightened risk of age-related diseases and mortality [16, 17]. However, the interrelation between CBC-derived inflammatory indicators and survival in sarcopenic individuals remains unexplored. Therefore, utilizing data extracted from the 1999–2006 National Health and Nutrition Examination Survey (NHANES), this study endeavors to elucidate the associations between CBC-derived inflammatory indicators, the prevalence of sarcopenia, and mortality in individuals with sarcopenia. By discerning distinctive patterns of CBC-derived inflammatory biomarker expression linked to adverse health outcomes in sarcopenic patients, we aspire to unravel the underlying mechanisms governing the processes of aging and disease, as well as identify novel targets for prevention and therapeutic interventions.

## Materials and methods

### Study population

Data for this study were obtained from the NHANES, a program administered by the Centers for Disease Control and Prevention (CDC) in the United States [S5]. Comprehensive health and nutritional information from a nationally representative cohort was collected through interviews, medical examinations, and laboratory assessments. Informed consent was obtained from all participants, and the research protocols were approved by the National Center for Health Statistics (NCHS) Research Ethics Review Board.

A total of 41,474 individuals from the NHANES 1999–2006 were included in this study. Exclusions comprised individuals with missing data on CBC parameters (*n* = 12,166), sarcopenia assessment data (*n* = 7,870), and those aged below 20 years (*n* = 8,749). Subsequently, among participants with sarcopenia, individuals without follow-up data were further excluded, resulting in the inclusion of 1,724 sarcopenic participants for survival analyses (Figure S1).

### Assessment of CBC-derived inflammatory indicators

Fasting venous blood samples were collected from all study participants to determine leukocyte, neutrophil, lymphocyte, and monocyte counts (expressed in 1000 cells/µL). The following CBC-derived inflammatory indicators were calculated: NLR, dNLR, MLR, NMLR, SIRI, and SII using the following formulas: NLR = neutrophil counts/lymphocyte counts, dNLR = neutrophil counts / (white blood cell counts - lymphocyte counts), MLR = monocyte counts/lymphocyte counts, NMLR = (monocyte counts + neutrophil counts) / lymphocyte counts, SIRI = neutrophil counts × monocyte counts / lymphocyte counts, and SII = platelet counts × neutrophil counts / lymphocyte counts [15, 18].

### Assessment of sarcopenia

Sarcopenia was defined using the sex-specific sarcopenia index cutoff values established by the National Institutes of Health (FNIH) (0.789 for males and 0.512 for females) [19]. Dual-energy X-ray absorptiometry (DXA) was used to measure the skeletal muscle mass of the extremities, and the sarcopenia index-defined as total appendicular skeletal muscle mass [in kg]/BMI [kg/m^2^] was computed. DXA on whole-body scans was performed using the Hologic Discovery Model Research Laboratory. DXA data for sarcopenia definition can be accessed from the NHANES website (https://wwwn.cdc.gov/Nchs/Nhanes/Dxa/Dxa.aspx).

### Assessment of mortality

Participants’ vital status was ascertained through linkage to the National Death Index (NDI), enabling the identification of deceased individuals. All-cause and cardiovascular mortality data were collected up to December 31, 2019, utilizing the 2019 Linked Mortality File (LMF), which represents the most current linkages between specific NCHS surveys and the NDI.

### Covariates

Fundamental participant characteristics, including age, sex, race, education level, and total energy intake, were obtained through interviews and laboratory assessments. Income was quantified using the poverty-income ratio (PIR), calculated by dividing household income by a factor specific to household size and composition [S6]. PIR was categorized into three groups: 1.0, 1.1-3.0, and > 3.0. Individual smoking and drinking status was recorded through a standardised questionnaire asking participants about their past and present smoking and drinking habits (cut-off value: 2 drinks/day in men and 1 drinks/day in women) [S7]. Physical activity levels were classified as inactive (no leisure-time physical activity), insufficiently active (moderate activity 1–5 times per week with metabolic equivalents [MET] 3–6 or vigorous activity 1–3 times per week with MET > 6), and active (individuals engaging in more moderate or vigorous activity than described above) [20, 21]. Data on diabetes and hypertension prevalence were collected through self-reported surveys.

### Statistical analysis

Continuous variables with non-normal distributions were evaluated using the Mann-Whitney U test and presented as medians (interquartile range [IQR]). Categorical variables were compared using the chi-square test and reported as counts (percentages). To approximate a normal distribution, a natural logarithm (ln) transformation was applied to continuous CBC-derived inflammatory indicators. Missing values for variables were imputed using the Random Forest algorithm’s “mice” package. All statistical analyses were conducted using R software (version 4.2.0), with statistical significance defined as a *P*-value < 0.05.

Multiple logistic regressions were employed to calculate adjusted odds ratios (ORs) and 95% confidence intervals (CIs) to assess the association between CBC-derived inflammatory indicators and the prevalence of sarcopenia. Multiple Cox regressions were utilized to determine adjusted hazard ratios (HRs) and 95% CIs for all-cause and cardiovascular mortality in sarcopenic subjects. The Benjamini-Hochberg (BH) method was employed to adjust for multiple testing and control the false discovery rate (FDR). Dose-response curves between CBC-derived inflammatory indicators and mortality in sarcopenic individuals were investigated using restricted cubic splines (RCS) with the 10th, 50th, and 90th percentiles as nodes.

Spearman’s correlation analysis was employed to compute correlation coefficients between CBC-derived inflammatory indicators and CBC parameters. The utility of CBC-derived inflammatory indicators in predicting all-cause and cardiovascular mortality in sarcopenia patients was compared using the random subsistence forest method. To mitigate potential reverse causality bias, additional Cox regression analyses were conducted after excluding cases where death occurred within the first two years of follow-up or patients with a history of cancer at baseline.

## Results

### Baseline characteristics of the study cohort

The baseline characteristics of the 12,689 participants are presented in Table 1. Of these, 1,725 individuals (13.59%) met the criteria for sarcopenia. The medians of the CBC-derived inflammatory indicators, including NLR, dNLR, MLR, NMLR, SIRI, and SII, were as follows: NLR 1.95 [1.48, 2.60], dNLR 1.42 [1.10, 1.80], MLR 0.26 [0.21, 0.33], NMLR 2.21 [0.71, 1.48], SIRI 1.02 [0.71, 1.48], and SII 509.22 [367.88, 707.44]. Participants with sarcopenia were more likely to be older males (> 59 years old), of Mexican American ethnicity, possess lower educational and income levels, be non-smokers and non-drinkers, exhibit sedentary behavior, and have a higher prevalence of hypertension and diabetes (*P* < 0.001). Among sarcopenic patients, there were significantly higher levels of white blood cells (WBC), neutrophils, monocytes, and other CBC-derived indicators (*P* < 0.001).

**Table 1:** Baseline characteristics of adults with CBC-derived inflammatory biomarkers in NHANES 1999–2006

Characteristics	Total (*n* = 12,689)		Sarcopenia		*P* value
			No (*n* = 10,964)	Yes (*n* = 1725)	
Age, years					< 0.001
<39	4449 (35.1)		4220 (38.5)	229 (13.3)	
40–59	4240 (33.4)		3822 (34.9)	418 (24.2)	
> 59	4000 (31.5)		2922 (26.7)	1078 (62.5)	
Male, %	6471 (51.0)		5537 (50.5)	934 (54.1)	0.005
Race/ethnicity, %					< 0.001
Mexican American	2951 (23.3)		2181 (19.9)	770 (44.6)	
Other Hispanic	568 (4.5)		469 (4.3)	99 (5.7)	
Non-Hispanic White	6389 (50.4)		5647 (51.5)	742 (43.0)	
Non-Hispanic Black	2329 (18.4)		2271 (20.7)	58 (3.4)	
Other race	452 (3.6)		396 (3.6)	56 (3.2)	
Education level, %					< 0.001
Below high school	3881 (30.6)		3017 (27.5)	864 (50.1)	
High school	3012 (23.7)		2631 (24.0)	381 (22.1)	
Above high school	5796 (45.7)		5316 (48.5)	480 (27.8)	
Family PIR, %					< 0.001
≤ 1.0	2240 (17.7)		1833 (16.7)	407 (23.6)	
1.1–3.0	5187 (40.9)		4326 (39.5)	861 (49.9)	
> 3.0	5262 (41.5)		4805 (43.8)	457 (26.5)	
Smoking status, %					< 0.001
Never smoker	6427 (50.7)		5574 (50.8)	853 (49.4)	
Former smoker	3246 (25.6)		2663 (24.3)	583 (33.8)	
Current smoker	3016 (23.8)		2727 (24.9)	289 (16.8)	
Drinking status, %					< 0.001
Nondrinker	2819 (22.2)		2291 (20.9)	528 (30.6)	
Low-to-moderate drinker	8740 (68.9)		7645 (69.7)	1095 (63.5)	
Heavy drinker	1130 (8.9)		1028 (9.4)	102 (5.9)	
Physical activity, %					< 0.001
Inactive	3396 (26.8)		2716 (24.8)	680 (39.4)	
Insufficiently active	6290 (49.6)		5563 (50.7)	727 (42.1)	
Active	3003 (23.7)		2685 (24.5)	318 (18.4)	
Total energy intakes, kcal/day	1962.00 [1438.00, 2643.00]		2017.49 [1480.45, 2712.83]	1656.00 [1223.79, 2194.10]	< 0.001
Self-reported hypertension, %	3727 (29.4)		2971 (27.1)	756 (43.8)	< 0.001
Self-reported diabetes, %	1150 (9.1)		830 (7.6)	320 (18.6)	< 0.001
CBC count, 10^3^/µL					
White blood cell	6.90 [5.70, 8.20]		6.80 [5.60, 8.20]	7.20 [6.10, 8.60]	< 0.001
Neutrophils	4.00 [3.10, 5.10]		3.90 [3.10, 5.00]	4.30 [3.40, 5.30]	< 0.001
Monocyte	0.50 [0.40, 0.60]		0.50 [0.40, 0.60]	0.60 [0.50, 0.70]	< 0.001
Lymphocyte	2.00 [1.60, 2.50]		2.00 [1.60, 2.50]	2.00 [1.60, 2.50]	0.466
CBC-derived indicators					
NLR	1.95 [1.48, 2.60]		1.93 [1.46, 2.56]	2.12 [1.60, 2.80]	< 0.001
dNLR	1.42 [1.10, 1.80]		1.40 [1.09, 1.79]	1.52 [1.18, 1.90]	< 0.001
MLR	0.26 [0.21, 0.33]		0.25 [0.20, 0.33]	0.27 [0.21, 0.36]	< 0.001
NMLR	2.21 [1.71, 2.91]		2.19 [1.69, 2.87]	2.40 [1.83, 3.15]	< 0.001
SIRI, 10^3^/µL	1.02 [0.71, 1.48]		1.00 [0.70, 1.44]	1.18 [0.80, 1.69]	< 0.001
SII, 10^3^/µL	509.22 [367.88, 707.44]		504.67 [364.50, 700.62]	546.54 [392.33, 763.82]	< 0.001

Abbreviations: PIR, poverty income ratio; NLR, neutrophil-to-lymphocyte ratio; dNLR, derived neutrophil-to-lymphocyte ratio; MLR, monocyte-to-lymphocyte ratio; NMLR, neutrophil-monocyte to lymphocyte ratio; SIRI, systemic inflammatory response index; SII, systemic immune-inflammation index; CBC, complete blood cell

Continuous variables without a normal distribution are presented as medians [interquartile ranges]. Categorical variables are presented as numbers (percentages). The Benjamini-Hochberg method was used to adjust *p* values for multiple testing

### Associations between CBC-Derived indicators and sarcopenia prevalence

In the crude model (Table 2), positive associations between CBC-derived indicators and sarcopenia prevalence were observed. After adjusting for confounding factors, these associations remained statistically significant, with the exception of MLR. In model 3, when compared to individuals in the lowest quartile of CBC-derived inflammatory indicators, those in the highest quartiles exhibited an elevated prevalence of sarcopenia (OR [95% CI]: 1.215 [1.037–1.425] for NLR, 1.177 [1.006–1.378] for dNLR, 1.226 [1.046–1.437] for NMLR, 1.397 [1.188–1.645] for SIRI, and 1.311 [1.122–1.533] for SII among quartile four participants of CBC-derived indicators). Additionally, we examined the relationship between CBC values and sarcopenia prevalence (Table S1) and found strong associations between WBC, neutrophil, and monocyte counts with a higher prevalence of sarcopenia in model 3.

**Table 2:** OR (95% CIs) of the prevalence of sarcopenia according to quartiles of complete blood cell (CBC)-derived inflammatory biomarkers among adults in NHANES 1999–2006

	Quartiles of CBC-derived inflammatory biomarkers levels				*P*_trend_
	Quartile 1	Quartile 2	Quartile 3	Quartile 4	
**NLR**					
Range	< 1.48	1.48–1.95	1.96–2.60	> 2.60	
Crude	1 [Reference]	1.194 (1.023–1.395)	1.450 (1.247–1.686)	1.748 (1.510–2.026)	< 0.001
Model 1	1 [Reference]	0.984 (0.835–1.159)	1.162 (0.990–1.364)	1.296 (1.107–1.518)	< 0.001
Model 2	1 [Reference]	0.985 (0.836–1.161)	1.157 (0.986–1.359)	1.215 (1.037–1.425)	0.004
**dNLR**					
Range	< 1.10	1.10–1.42	1.43–1.80	> 1.80	
Crude	1 [Reference]	1.186 (1.017–1.383)	1.397 (1.204–1.623)	1.620 (1.400-1.877)	< 0.001
Model 1	1 [Reference]	0.998 (0.849–1.175)	1.150 (0.982–1.349)	1.261 (1.079–1.475)	< 0.001
Model 2	1 [Reference]	0.971 (0.825–1.143)	1.159 (0.988–1.359)	1.177 (1.006–1.378)	0.008
**MLR**					
Range	< 0.21	0.21–0.26	0.27–0.33	> 0.33	
Crude	1 [Reference]	0.979 (0.842–1.139)	1.073 (0.924–1.247)	1.408 (1.225–1.618)	< 0.001
Model 1	1 [Reference]	0.840 (0.715–0.987)	0.842 (0.716–0.991)	1.048 (0.896–1.226)	0.381
Model 2	1 [Reference]	0.823 (0.700-0.968)	0.822 (0.699–0.967)	1.004 (0.857–1.176)	0.705
**NMLR**					
Range	< 1.71	1.71–2.21	2.22–2.91	> 2.91	
Crude	1 [Reference]	1.219 (1.044–1.423)	1.411 (1.213–1.642)	1.766 (1.527–2.045)	< 0.001
Model 1	1 [Reference]	1.006 (0.854–1.185)	1.142 (0.973–1.342)	1.305 (1.115–1.529)	< 0.001
Model 2	1 [Reference]	1.001 (0.850–1.180)	1.140 (0.971–1.340)	1.226 (1.046–1.437)	0.004
**SIRI**					
Range	< 0.71	0.71–1.02	1.03–1.48	> 1.48	
Crude	1 [Reference]	1.314 (1.123–1.538)	1.523 (1.306–1.776)	1.997 (1.723–2.317)	< 0.001
Model 1	1 [Reference]	1.046 (0.886–1.235)	1.184 (1.005–1.395)	1.525 (1.299–1.792)	< 0.001
Model 2	1 [Reference]	1.016 (0.861–1.201)	1.138 (0.966–1.342)	1.397 (1.188–1.645)	< 0.001
**SII**					
Range	< 367.88	367.88-509.22	509.23-7707.44	> 707.44	
Crude	1 [Reference]	1.155 (0.993–1.343)	1.309 (1.129–1.518)	1.470 (1.271-1.700)	< 0.001
Model 1	1 [Reference]	0.999 (0.852–1.173)	1.218 (1.041–1.425)	1.357 (1.163–1.585)	< 0.001
Model 2	1 [Reference]	1.001 (0.853–1.176)	1.226 (1.047–1.437)	1.311 (1.122–1.533)	< 0.001

The Benjamini-Hochberg method was used to adjust *p* values for multiple testing. Model 1 was adjusted as age (< 39, 40–59, or > 59), sex (male or female), and race/ethnicity (Mexican American, Other Hispanic, Non-Hispanic White, Non-Hispanic Black or Other); Model 2 was adjusted as model 1 plus education level (below high school, high school, or above high school), family poverty income ratio (≤ 1.0, 1.1–3.0, or > 3.0), drinking status (nondrinker, low-to-moderate drinker, or heavy drinker), smoking status (never smoker, former smoker, or current smoker), physical activity (inactive, insufficiently active, or active), total energy intakes (in quartiles), self-reported diabetes (yes or no), and self-reported hypertension (yes or no)

### Associations between CBC-Derived indicators and all-cause mortality among adults with Sarcopenia

**Fig. 1:**
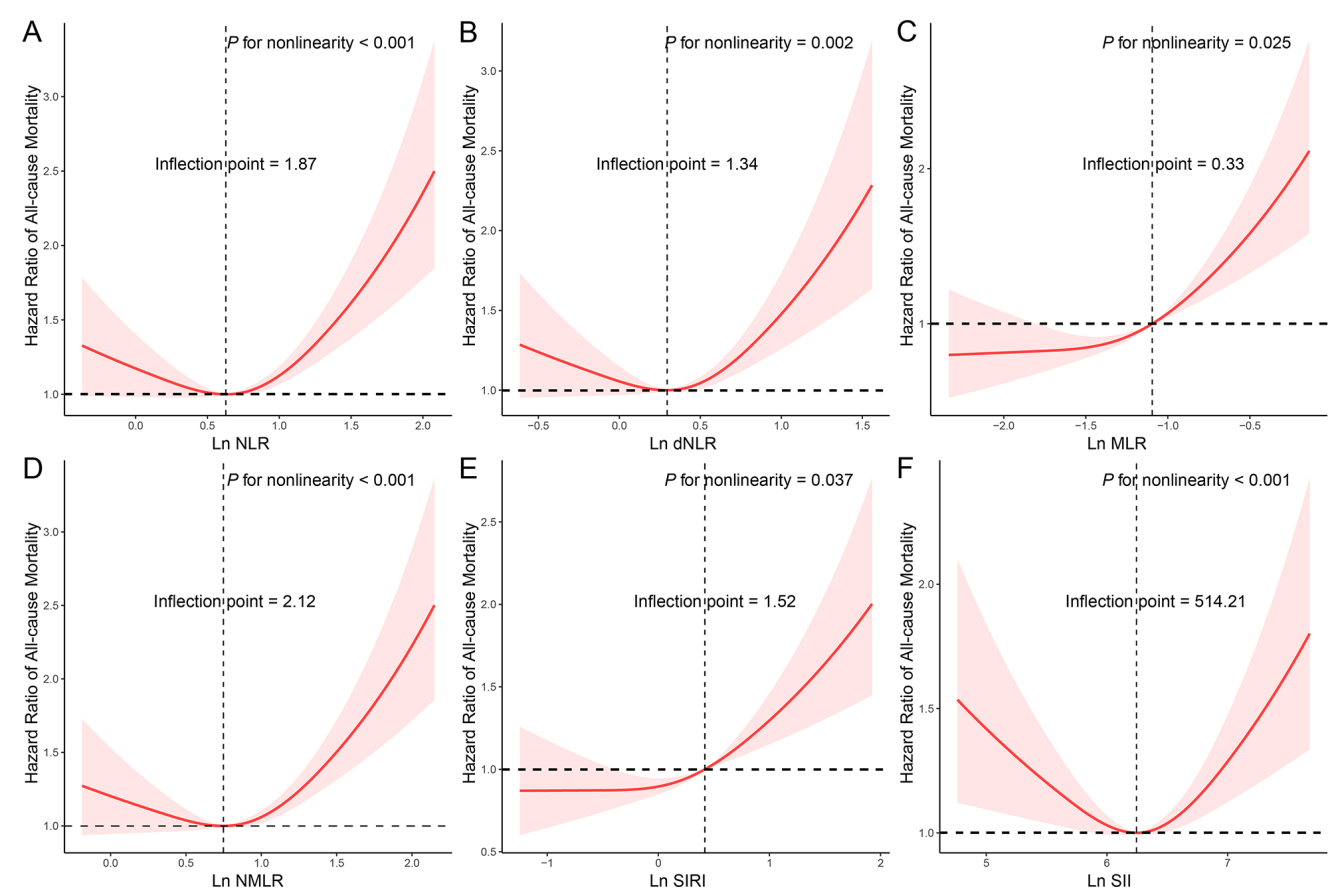
Application of restricted cubic spline (RCS) regression to examine the association between complete blood cell (CBC)-derived indicators and all-cause mortality in the adult sarcopenic population. Model was adjusted as age (< 39, 40–59, or > 59), sex (male or female), race/ethnicity (Mexican American, Other Hispanic, Non-Hispanic White, Non-Hispanic Black or Other), education level (below high school, high school, or above high school), family poverty income ratio (≤ 1.0, 1.1–3.0, or > 3.0), drinking status (nondrinker, low-to-moderate drinker, or heavy drinker), smoking status (never smoker, former smoker, or current smoker), physical activity (inactive, insufficiently active, or active), total energy intakes (in quartiles), self-reported diabetes (yes or no), and self-reported hypertension (yes or no)

During a median follow-up period of 14.67 [9.67, 17.58] years, 782 (44.89%) all-cause deaths occurred among the 1,742 adults with sarcopenia (Table S2). Deceased individuals exhibited higher levels of CBC-derived indicators compared to survivors (*P* < 0.001). Except for SII, patients with sarcopenia in quartile four of CBC-derived indicators had the highest risk of all-cause mortality in the crude model, as indicated in Table 3. These associations remained stable in model 1. Following adjustment for all covariates in the full model, individuals in the highest quartile of NLR (HR = 1.336 [1.095–1.631]), dNLR (HR = 1.274 [1.046–1.550]), MLR (HR = 1.619 [1.290–2.032]), NMLR (HR = 1.390 [1.132–1.707]), and SIRI (HR = 1.501 [1.210–1.862]) were associated with an increased risk of all-cause mortality compared to those in the lowest quartile of inflammation-derived indicators. Figure 1 illustrates the non-linear associations between CBC-derived indicators (including NLR, dNLR, MLR, NMLR, SIRI, and SII) and all-cause mortality among sarcopenic participants, with inflection points of 1.87, 1.34, 0.33, 2.21, 1.52, and 514.21, respectively (all *P* for nonlinearity < 0.05). Furthermore, after accounting for all confounding variables, monocyte count was associated with an increased risk of all-cause mortality (HR = 1.232 [1.010–1.502]), while lymphocyte count exhibited an inverse relationship (HR = 0.715 [0.581–0.881]) (Table S3).

**Table 3:** HRs (95% CIs) of all-cause mortality according to quartiles of complete blood cell (CBC)-derived inflammatory biomarkers among adults with sarcopenia in NHANES 1999–2006

	Quartiles of CBC-derived inflammatory biomarkers levels				*P*_trend_
	Quartile 1	Quartile 2	Quartile 3	Quartile 4	
**NLR**					
Range	< 1.60	1.60–2.12	2.13–2.80	> 2.80	
No. deaths/total	174/437	160/427	186/429	262/431	
Crude	1 [Reference]	0.909 (0.733–1.126)	1.118 (0.909–1.375)	1.908 (1.575–2.311)	< 0.001
Model 1	1 [Reference]	0.842 (0.678–1.046)	1.010 (0.819–1.246)	1.391 (1.141–1.695)	< 0.001
Model 2	1 [Reference]	0.830 (0.667–1.032)	0.950 (0.768–1.175)	1.336 (1.095–1.631)	< 0.001
**dNLR**					
Range	< 1.18	1.18–1.52	1.53–1.90	> 1.90	
No. deaths/total	188/438	162/424	196/433	236/429	
Crude	1 [Reference]	0.839 (0.680–1.036)	1.063 (0.871–1.299)	1.475 (1.218–1.787)	< 0.001
Model 1	1 [Reference]	0.833 (0.674–1.030)	0.990 (0.809–1.212)	1.326 (1.092–1.611)	< 0.001
Model 2	1 [Reference]	0.784 (0.633–0.971)	0.934 (0.761–1.146)	1.274 (1.046–1.550)	0.004
**MLR**					
Range	< 0.21	0.21–0.27	0.28–0.36	> 0.36	
No. deaths/total	133/440	176/453	191/386	292/445	
Crude	1 [Reference]	1.339 (1.069–1.677)	1.831 (1.467–2.284)	2.961 (2.409–3.640)	< 0.001
Model 1	1 [Reference]	1.028 (0.817–1.294)	1.186 (0.942–1.494)	1.572 (1.257–1.967)	< 0.001
Model 2	1 [Reference]	1.085 (0.861–1.368)	1.178 (0.933–1.487)	1.619 (1.290–2.032)	< 0.001
**NMLR**					
Range	< 1.83	1.83–2.40	2.41–3.15	> 3.15	
No. deaths/total	161/426	165/437	195/430	261/431	
Crude	1 [Reference]	0.978 (0.787–1.215)	1.273 (1.033–1.568)	2.045 (1.680–2.490)	< 0.001
Model 1	1 [Reference]	0.926 (0.744–1.152)	1.108 (0.896–1.370)	1.450 (1.183–1.777)	< 0.001
Model 2	1 [Reference]	0.929 (0.745–1.157)	1.040 (0.839–1.290)	1.390 (1.132–1.707)	< 0.001
**SIRI**					
Range	< 0.80	0.80–1.18	1.18–1.69	> 1.69	
No. deaths/total	148/440	155/422	219/430	260/432	
Crude	1 [Reference]	1.116 (0.891–1.398)	1.776 (1.441–2.188)	2.320 (1.896–2.840)	< 0.001
Model 1	1 [Reference]	1.049 (0.836–1.317)	1.390 (1.121–1.723)	1.589 (1.284–1.968)	< 0.001
Model 2	1 [Reference]	1.050 (0.835–1.319)	1.313 (1.058–1.631)	1.501 (1.210–1.862)	< 0.001
**SII**					
Range	< 392.25	392.25-546.75	546.76-764.06	> 764.06	
No. deaths/total	201/431	190/431	180/431	211/431	
Crude	1 [Reference]	0.907 (0.744–1.106)	0.879 (0.719–1.075)	1.090 (0.899–1.323)	0.455
Model 1	1 [Reference]	0.875 (0.717–1.068)	0.918 (0.749–1.125)	1.092 (0.898–1.328)	0.350
Model 2	1 [Reference]	0.872 (0.714–1.065)	0.901 (0.735–1.105)	1.073 (0.881–1.307)	0.455

The Benjamini-Hochberg method was used to adjust *p* values for multiple testing. Model 1 was adjusted as age (< 39, 40–59, or > 59), sex (male or female), and race/ethnicity (Mexican American, Other Hispanic, Non-Hispanic White, Non-Hispanic Black or Other); Model 2 was adjusted as model 1 plus education level (below high school, high school, or above high school), family poverty income ratio (≤ 1.0, 1.1–3.0, or > 3.0), drinking status (nondrinker, low-to-moderate drinker, or heavy drinker), smoking status (never smoker, former smoker, or current smoker), physical activity (inactive, insufficiently active, or active), total energy intakes (in quartiles), self-reported diabetes (yes or no), and self-reported hypertension (yes or no)

### Associations between CBC-Derived indicators and Cardiovascular Mortality among adults with Sarcopenia

**Fig. 2:**
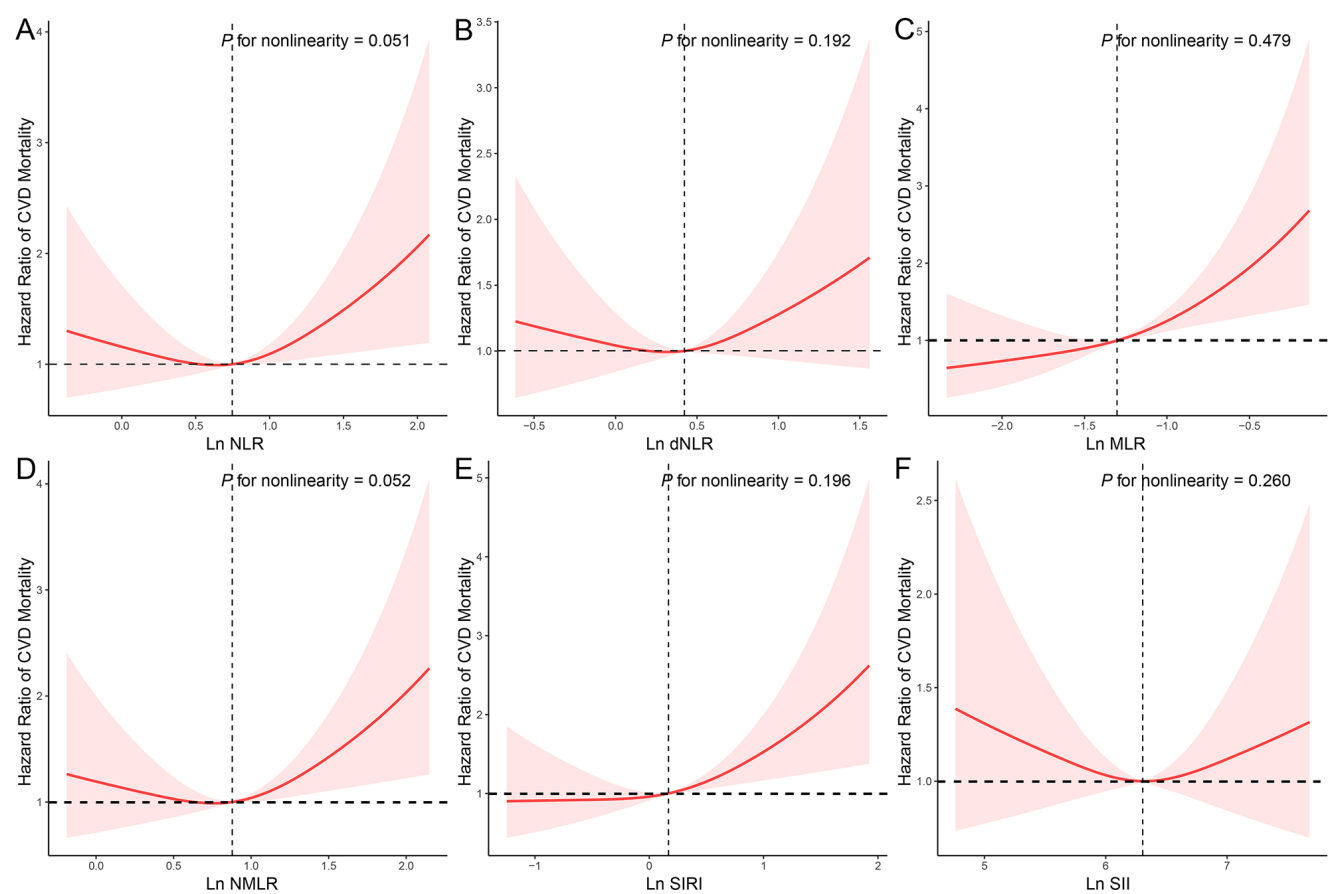
Application of restricted cubic spline (RCS) regression to examine the association between complete blood cell (CBC)-derived indicators and cardiovascular mortality in the adult sarcopenic population. Model was adjusted as age (< 39, 40–59, or > 59), sex (male or female), race/ethnicity (Mexican American, Other Hispanic, Non-Hispanic White, Non-Hispanic Black or Other), education level (below high school, high school, or above high school), family poverty income ratio (≤ 1.0, 1.1–3.0, or > 3.0), drinking status (nondrinker, low-to-moderate drinker, or heavy drinker), smoking status (never smoker, former smoker, or current smoker), physical activity (inactive, insufficiently active, or active), total energy intakes (in quartiles), self-reported diabetes (yes or no), and self-reported hypertension (yes or no)

During the follow-up period, 195 (11.19%) of the 1,742 adults with sarcopenia experienced cardiovascular deaths. Cox proportional regression analyses assessed the associations of CBC-derived indicators with cardiovascular mortality in sarcopenia patients (Table 4). The results indicated that, in the crude model, NLR, MLR, NMLR, and SIRI were linked to an elevated risk of cardiovascular mortality. In model 1, only MLR and SIRI retained these associations. Following full model adjustment, the highest quartile of MLR (HR = 1.874 [1.169–3.003]) and SIRI (HR = 1.838 [1.175–2.878]) were associated with an increased risk of cardiovascular mortality compared to individuals in the lowest quartile of inflammation-derived indicators. Figure 2 demonstrates the non-linear associations between MLR and SIRI with cardiovascular mortality among adults with sarcopenia (all *P* for nonlinearity < 0.05). The relationship between CBC values and the risk of cardiovascular death in individuals with sarcopenia was also examined (Table S4), revealing that only monocyte count was associated with an increased likelihood of cardiovascular mortality (HR = 1.694 [1.163–2.466]), after adjusting for all covariates.

**Table 4:** HRs (95% CIs) of cardiovascular mortality according to quartiles of complete blood cell (CBC)-derived inflammatory biomarkers among adults with sarcopenia in NHANES 1999–2006

	Quartiles of CBC-derived inflammatory biomarkers levels				*P*_trend_
	Quartile 1	Quartile 2	Quartile 3	Quartile 4	
**NLR**					
Range	< 1.60	1.60–2.12	2.13–2.80	> 2.80	
No. deaths/total	39/437	45/427	46/429	65/431	
Crude	1 [Reference]	1.148 (0.747–1.762)	1.231 (0.804–1.887)	2.085 (1.402–3.103)	< 0.001
Model 1	1 [Reference]	1.038 (0.674–1.598)	1.057 (0.686–1.629)	1.407 (0.936–2.116)	0.144
Model 2	1 [Reference]	1.011 (0.654–1.564)	0.949 (0.613–1.470)	1.301 (0.863–1.962)	0.295
**dNLR**					
Range	< 1.18	1.18–1.52	1.53–1.90	> 1.90	
No. deaths/total	44/438	46/424	50/433	55/429	
Crude	1 [Reference]	1.027 (0.679–1.552)	1.158 (0.772–1.737)	1.460 (0.982–2.171)	0.090
Model 1	1 [Reference]	1.003 (0.661–1.522)	1.038 (0.689–1.564)	1.250 (0.836–1.868)	0.343
Model 2	1 [Reference]	0.917 (0.600-1.401)	0.942 (0.622–1.427)	1.157 (0.771–1.737)	0.470
**MLR**					
Range	< 0.21	0.21–0.27	0.28–0.36	> 0.36	
No. deaths/total	28/440	41/453	46/386	80/445	
Crude	1 [Reference]	1.477 (0.913–2.388)	2.078 (1.299–3.324)	3.902 (2.536–6.005)	< 0.001
Model 1	1 [Reference]	1.062 (0.651–1.732)	1.227 (0.753–1.998)	1.842 (1.158–2.932)	0.006
Model 2	1 [Reference]	1.123 (0.685–1.841)	1.234 (0.754–2.022)	1.874 (1.169–3.003)	0.008
**NMLR**					
Range	< 1.83	1.83–2.40	2.41–3.15	> 3.15	
No. deaths/total	36/426	45/437	47/430	67/431	
Crude	1 [Reference]	1.200 (0.774–1.859)	1.364 (0.884–2.105)	2.314 (1.543–3.471)	< 0.001
Model 1	1 [Reference]	1.110 (0.714–1.725)	1.122 (0.722–1.742)	1.506 (0.992–2.287)	0.090
Model 2	1 [Reference]	1.118 (0.717–1.743)	1.003 (0.642–1.567)	1.388 (0.911–2.114)	0.231
**SIRI**					
Range	< 0.80	0.80–1.18	1.18–1.69	> 1.69	
No. deaths/total	30/440	40/422	49/430	76/432	
Crude	1 [Reference]	1.419 (0.884–2.278)	1.933 (1.227–3.046)	3.292 (2.156–5.025)	< 0.001
Model 1	1 [Reference]	1.294 (0.802–2.088)	1.409 (0.883–2.249)	2.053 (1.315–3.205)	< 0.001
Model 2	1 [Reference]	1.285 (0.795–2.078)	1.272 (0.796–2.032)	1.838 (1.175–2.878)	0.016
**SII**					
Range	< 392.25	392.25-546.75	546.76-764.06	> 764.06	
No. deaths/total	57/431	48/431	40/431	50/431	
Crude	1 [Reference]	0.810 (0.552–1.190)	0.693 (0.462–1.038)	0.911 (0.623–1.331)	0.470
Model 1	1 [Reference]	0.767 (0.521–1.127)	0.706 (0.469–1.061)	0.882 (0.600-1.295)	0.470
Model 2	1 [Reference]	0.770 (0.522–1.136)	0.693 (0.460–1.044)	0.845 (0.573–1.246)	0.400

The Benjamini-Hochberg method was used to adjust *p* values for multiple testing. Model 1 was adjusted as age (< 39, 40–59, or > 59), sex (male or female), and race/ethnicity (Mexican American, Other Hispanic, Non-Hispanic White, Non-Hispanic Black or Other); Model 2 was adjusted as model 1 plus education level (below high school, high school, or above high school), family poverty income ratio (≤ 1.0, 1.1–3.0, or > 3.0), drinking status (nondrinker, low-to-moderate drinker, or heavy drinker), smoking status (never smoker, former smoker, or current smoker), physical activity (inactive, insufficiently active, or active), total energy intakes (in quartiles), self-reported diabetes (yes or no), and self-reported hypertension (yes or no)

### Prognostic value of CBC-Derived indicators

**Fig. 3:**
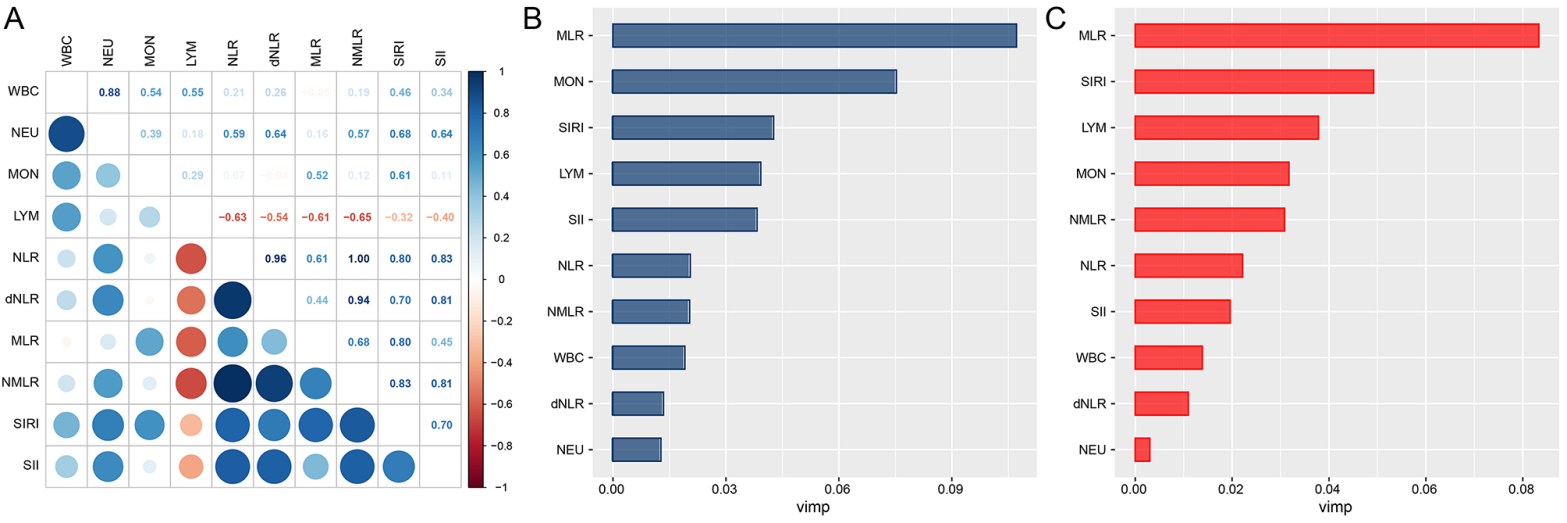
Prognostic value of complete blood cell (CBC)-derived indicators. Spearman correlation analysis was used to calculate the correlation coefficients among CBC parameters and CBC-derived inflammatory indicators (**A**). The random survival forests (RSF) method was used to compare the value of CBC parameters and CBC-derived inflammatory indicators in predicting all-cause mortality (**B**) and cardiovascular mortality (**C**) among adults with sarcopenia. Model was adjusted as age (< 39, 40–59, or > 59), sex (male or female), race/ethnicity (Mexican American, Other Hispanic, Non-Hispanic White, Non-Hispanic Black or Other), education level (below high school, high school, or above high school), family poverty income ratio (≤ 1.0, 1.1–3.0, or > 3.0), drinking status (nondrinker, low-to-moderate drinker, or heavy drinker), smoking status (never smoker, former smoker, or current smoker), physical activity (inactive, insufficiently active, or active), total energy intakes (in quartiles), self-reported diabetes (yes or no), and self-reported hypertension (yes or no)

The correlation between CBC parameters and CBC-derived inflammatory indicators is presented in Fig. 3A. Notably, a strong positive correlation was observed between NLR and NMLR (*r* = 0.995), while a substantial negative correlation was found between lymphocyte count and NMLR (*r*=-0.654). Figure 3B and C reveal that, among all CBC inflammatory indicators and CBC-derived inflammatory indicators, MLR exhibited the highest predictive value for all-cause and cardiovascular mortality in adults with sarcopenia.

### Sensitivity analyses

Sensitivity analyses were conducted to address potential reverse causality bias by excluding individuals who died within the first two years of follow-up (Table S5). Notably, these analyses confirmed the persistence of associations between MLR, NMLR, and SIRI with outcomes. Similarly, after excluding participants with a history of cancer at baseline, our findings remained consistent (Table S6).

## Discussion

Our study revealed favorable associations between NLR, dNLR, NMLR, SIRI, and SII with sarcopenia prevalence using data from the NHANES 1999–2006. In adults with sarcopenia, NLR, dNMLR, MLR, and SIRI displayed non-linear and robust associations with the risk of all-cause mortality. Participants in the highest quartiles of MLR and SIRI had an increased risk of cardiovascular mortality. Furthermore, MLR emerged as the most potent predictor of both all-cause and cardiovascular mortality in individuals with sarcopenia.

As the global population continues to age, the health of older adults has become a growing concern [22]. Among the myriad health challenges faced by the elderly, sarcopenia and mortality stand out as significant issues [23, 24]. Recent research has pointed towards inflammation as a contributing factor to the development of sarcopenia [25]. Hence, it is imperative to identify inflammatory markers associated with the risk of sarcopenia and mortality.

Our study found that WBC, neutrophil, and monocyte counts were significantly linked to a higher prevalence of sarcopenia. Specifically, monocytes were significantly associated with an increased risk of all-cause and cardiovascular mortality in sarcopenic individuals. These findings are in line with previous research that highlighted the role of various blood cell counts in the context of sarcopenia. For example, Lee et al. demonstrated that higher platelet and WBC counts were independently associated with sarcopenia in Korean adults [26]. Additionally, Gholizade et al. identified a link between platelet to WBC ratio (PWR) and sarcopenia [27]. Another study in Korea found that elevated WBC counts were independently associated with sarcopenia in older men [28]. Similarly, a positive correlation between WBC counts and sarcopenia risk was observed in postmenopausal women [29].

CBC is a widely employed laboratory test for quantifying various blood cell types. A growing body of research has explored the relationship between CBC-derived inflammatory markers and the risk of chronic diseases and mortality. Ke et al. found that NLR, PLR, MLR, SIRI, and SII were associated with the prevalence of asthma and increased the risk of all-cause and respiratory disease mortality in asthma patients [14]. Shoji et al. reported that high MLR levels were linked to poorer five-year recurrence-free survival rates [15]. Additionally, other studies have demonstrated associations between CBC-derived markers and various health conditions, including peritoneal dialysis-associated peritonitis [18], disease activity in rheumatoid arthritis, and the prognosis of renal involvement in systemic lupus erythematosus [30]. Furthermore, NLR has been identified as a prognostic indicator for cardiovascular events in patients with coronary artery disease [31]. Multiple investigations have found that NLR predicts disease progression and overall survival in patients with prostate cancer [32]. NLR, dNLR, NMLR, SIRI, and SII all demonstrated significant associations with the prevalence of sarcopenia in our study. Moreover, MLR and SIRI were associated with an elevated risk of all-cause and cardiovascular mortality in sarcopenic patients. The random survival forest analysis identified MLR as the most robust predictor of all-cause and cardiovascular mortality in adults with sarcopenia.

Sarcopenia is a complex multifactorial condition characterized by muscle wasting and a decline in skeletal muscle mass [33]. While the exact pathogenesis of sarcopenia remains elusive, recent research suggests that the interplay between immune cells and inflammation may play a pivotal role in its development [34, 35]. WBCs are integral components of the immune system and are involved in modulating inflammation. Their immunological activity can accelerate the onset of sarcopenia by increasing oxidative stress, enhancing cytokine release, and causing muscle fiber damage through the generation of free radicals and reactive oxygen species [36, 37]. Additionally, inflammation can impact muscle tissue metabolism, leading to insulin resistance and metabolic syndrome, both of which are risk factors for sarcopenia [38]. Inflammation can also disrupt muscle protein synthesis and function, resulting in muscle weakness and atrophy [39]. Beyond immune cells, factors such as genetics and environmental toxins have also been implicated in sarcopenia development [40]. However, the precise involvement of inflammation in this process remains incompletely understood, necessitating further research to unravel the intricate interplay between immune cells and sarcopenia.

In summary, our study presents several notable strengths. Firstly, the robustness of our findings is fortified by the substantial sample size, incorporating a comprehensive cohort of 12,689 participants, thereby augmenting the applicability and relevance of our results. Secondly, it represents a pioneering investigation that delves into the intricate association between CBC-derived markers and mortality within the sarcopenic population across an extended follow-up duration. Thirdly, the utilization of CBC as a readily available and cost-effective laboratory test contributes to the wealth of data accessible for extensive-scale investigations. Lastly, by employing the RSF method, which remains unaffected by the collinearity among strongly correlated inflammatory markers, our study methodically identified the most potent prognostic indicator among all CBC-related inflammatory markers—MLR. This approach transcends the limitations associated with single indices and underscores the predictive value of CBC-derived inflammatory indicators.

Nevertheless, this study is not without its limitations, which merit consideration and further investigation. Firstly, our study draws upon data exclusively from the NHANES database, thus primarily reflecting trends within the United States population. Consequently, the generalizability of our findings to other global populations may be constrained. Secondly, despite meticulous adjustments for confounding factors, the presence of unmeasured variables remains a potential source of influence on the analytical outcomes. The complex interplay of various factors involved in sarcopenia and its associated mortality warrants a comprehensive exploration in future research endeavors. Thirdly, the computation of CBC-derived markers in our study relied on single-time CBC measurements, which, while practical, may introduce a degree of variability and potential bias into the analytical framework. Longitudinal assessments and repeated measurements could provide more robust insights into the dynamic nature of these markers in relation to sarcopenia and mortality.

## Conclusions

Our study demonstrates a clear association between elevated inflammatory status, as indicated by CBC-derived markers, and an increased prevalence of sarcopenia as well as a higher risk of mortality in sarcopenic individuals. These findings underscore the potential significance of monitoring CBC-derived inflammatory indicators as potential biomarkers in the context of sarcopenia. Further prospective investigations are needed to validate and expand upon these associations. By elucidating the role of CBC-derived inflammatory indicators in the development of sarcopenia, we aim to contribute valuable insights that can inform targeted interventions aimed at promoting healthy aging and reducing the burden of chronic illnesses in older populations.

Contributors BG was responsible for the investigation and writing original draft, organization and coordination of the trial. XL and QS also was one of the chief investigator. XL and DZ was responsible for methodolgy. YZ and ML was responsible for the data validation, while YW and MZ mainly conduct software practical operations. BG, FH and DH supervised the this project. All authors contributed to the writing of the final manuscript. All authors reviewed the manuscript.

### Electronic supplementary material

Below is the link to the electronic supplementary material.

Supplementary Material 1

Supplementary Material 2

Supplementary Material 3

## Data Availability

The datasets used and/or analyzed during the current study are available from the corresponding author on reasonable request. The detailed information on the data is available at https://wwwn.cdc.gov/nchs/nhanes/.
