# Supplementary material for: Associations of CBC-Derived inflammatory indicators with sarcopenia and mortality in adults: evidence from Nhanes 1999 ∼ 2006
Source: BMC Geriatr. 2024 May 16;24:432. doi: 10.1186/s12877-024-05012-2 (PMC11100216; doi:10.1186/s12877-024-05012-2)
Supplement: Supplementary file 3 — Supplementary Material 3 [file 12877_2024_5012_MOESM3_ESM.docx]

**Online Supplementary Material**

**
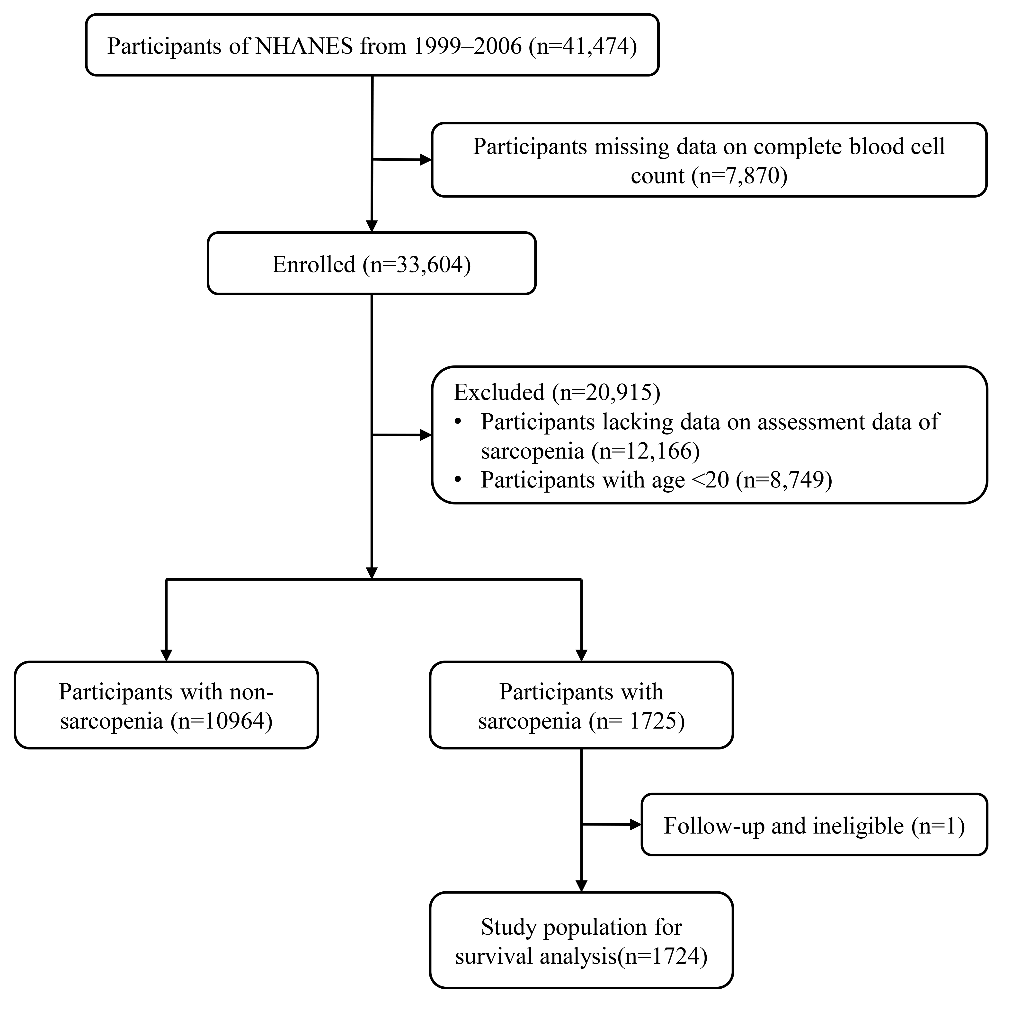
**

**Figure S1.** Flowchart of the study participants

**Table S1.** OR (95% CIs) of the prevalence of sarcopenia according to quartiles of complete blood cell (CBC) counts among adults in NHANES 1999–2006.

|  | Quartiles of CBC-derived inflammatory biomarkers levels | | | | *P*_trend_ |
| --- | --- | --- | --- | --- | --- |
|  | Quartile 1 | Quartile 2 | Quartile 3 | Quartile 4 |  |
| **WBC** | |  |  |  |  |
| Crude | 1 [Reference] | 1.491 (1.279-1.739) | 1.724 (1.480-2.010) | 1.901 (1.639-2.208) | <0.001 |
| Model 1 | 1 [Reference] | 1.291 (1.100- 1.516) | 1.548 (1.319- 1.818) | 1.871 (1.600- 2.191) | <0.001 |
| Model 2 | 1 [Reference] | 1.200 (1.022-1.409) | 1.453 (1.236-1.708) | 1.725 (1.469-2.028) | <0.001 |
| **NEU** | |  |  |  |  |
| Crude | 1 [Reference] | 1.665 (1.424-1.949) | 1.911 (1.640-2.231) | 2.133 (1.829-2.491) | <0.001 |
| Model 1 | 1 [Reference] | 1.347 (1.143-1.589) | 1.527 (1.300-1.798) | 1.937 (1.646-2.282) | <0.001 |
| Model 2 | 1 [Reference] | 1.268 (1.076-1.496) | 1.430 (1.216-1.685) | 1.768 (1.497-2.090) | <0.001 |
| **MON** |  |  |  |  |  |
| Crude | 1 [Reference] | 1.290 (1.118-1.488) | 1.340 (1.152-1.558) | 1.538 (1.337-1.768) | <0.001 |
| Model 1 | 1 [Reference] | 1.207 (1.038-1.403) | 1.235 (1.052-1.449) | 1.433 (1.233-1.665) | <0.001 |
| Model 2 | 1 [Reference] | 1.175 (1.010-1.367) | 1.176 (1.001-1.382) | 1.338 (1.150-1.557) | <0.001 |
| **LYM** |  |  |  |  |  |
| Crude | 1 [Reference] | 0.870 (0.755-1.003) | 0.968 (0.843-1.112) | 0.909 (0.786-1.051) | 0.431 |
| Model 1 | 1 [Reference] | 1.004 (0.864-1.166) | 1.228 (1.059-1.425) | 1.164 (0.995-1.361) | 0.010 |
| Model 2 | 1 [Reference] | 1.040 (0.894-1.211) | 1.241 (1.068-1.443) | 1.146 (0.977-1.343) | 0.019 |

The Benjamini-Hochberg method was used to adjust p values for multiple testing. Model 1 was adjusted as age (<39, 40-59, or >59), sex (male or female), and race/ethnicity (Mexican American, Other Hispanic, Non-Hispanic White, Non-Hispanic Black or Other); Model 2 was adjusted as model 1 plus education level (below high school, high school, or above high school), family poverty income ratio (≤1.0, 1.1–3.0, or >3.0), drinking status (nondrinker, low-to-moderate drinker, or heavy drinker), smoking status (never smoker, former smoker, or current smoker), physical activity (inactive, insufficiently active, or active), total energy intakes (in quartiles), self-reported diabetes (yes or no), and self-reported hypertension (yes or no).

**Table S2.** Baseline characteristics of adults with sarcopenia in NHANES 1999–2006.

| Characteristics | Total (n= 1724) | | All-cause Mortality | | *P* value |
| --- | --- | --- | --- | --- | --- |
|  |  |  | No (n= 942) | Yes (n= 782) |  |
| Age, years |  | |  |  | <0.001 |
| <39 | 229 (13.3) | | 221 (23.5) | 8 (1.0) |  |
| 40-59 | 417 (24.2) | | 334 (35.5) | 83 (10.6) |  |
| >59 | 1078 (62.5) | | 387 (41.1) | 691 (88.4) |  |
| Male, % | 933 (54.1) | | 457 (48.5) | 476 (60.9) | <0.001 |
| Race/ethnicity, % |  | |  |  | <0.001 |
| Mexican American | 770 (44.7) | | 524 (55.6) | 246 (31.5) |  |
| Other Hispanic | 99 (5.7) | | 62 (6.6) | 37 (4.7) |  |
| Non-Hispanic White | 741 (43.0) | | 292 (31.0) | 449 (57.4) |  |
| Non-Hispanic Black | 58 (3.4) | | 27 (2.9) | 31 (4.0) |  |
| Other race | 56 (3.2) | | 37 (3.9) | 19 (2.4) |  |
| Education level, % |  | |  |  | 0.331 |
| Below high school | 864 (50.1) | | 484 (51.4) | 380 (48.6) |  |
| High school | 381 (22.1) | | 195 (20.7) | 186 (23.8) |  |
| Above high school | 479 (27.8) | | 263 (27.9) | 216 (27.6) |  |
| Family PIR, % |  | |  |  | 0.001 |
| ≤1.0 | 407 (23.6) | | 248 (26.3) | 159 (20.3) |  |
| 1.1–3.0 | 860 (49.9) | | 431 (45.8) | 429 (54.9) |  |
| >3.0 | 457 (26.5) | | 263 (27.9) | 194 (24.8) |  |
| Smoking status, % |  | |  |  | <0.001 |
| Never smoker | 853 (49.5) | | 548 (58.2) | 305 (39.0) |  |
| Former smoker | 582 (33.8) | | 245 (26.0) | 337 (43.1) |  |
| Current smoker | 289 (16.8) | | 149 (15.8) | 140 (17.9) |  |
| Drinking status, % |  | |  |  | 0.637 |
| Nondrinker | 528 (30.6) | | 295 (31.3) | 233 (29.8) |  |
| Low-to-moderate drinker | 1094 (63.5) | | 595 (63.2) | 499 (63.8) |  |
| Heavy drinker | 102 (5.9) | | 52 (5.5) | 50 (6.4) |  |
| Physical activity, % |  | |  |  | 0.004 |
| Inactive | 679 (39.4) | | 341 (36.2) | 338 (43.2) |  |
| Insufficiently active | 727 (42.2) | | 430 (45.6) | 297 (38.0) |  |
| Active | 318 (18.4) | | 171 (18.2) | 147 (18.8) |  |
| Total energy intakes, kcal/day | 1655.72 [1223.09, 2194.06] | | 1751.50 [1296.25, 2279.24] | 1555.57 [1147.25, 2079.00] | <0.001 |
| Self-reported hypertension, % | 755 (43.8) | | 327 (34.7) | 428 (54.7) | <0.001 |
| Self-reported diabetes, % | 320 (18.6) | | 127 (13.5) | 193 (24.7) | <0.001 |
| CBC count, 10^3^/μL |  | |  |  |  |
| White blood cell | 7.20 [6.10, 8.60] | | 7.30 [6.10, 8.60] | 7.10 [6.10, 8.60] | 0.432 |
| Neutrophils | 4.30 [3.40, 5.30] | | 4.30 [3.40, 5.30] | 4.30 [3.50, 5.40] | 0.261 |
| Monocyte | 0.60 [0.50, 0.70] | | 0.50 [0.40, 0.60] | 0.60 [0.50, 0.70] | <0.001 |
| Lymphocyte | 2.00 [1.60, 2.50] | | 2.10 [1.70, 2.60] | 1.90 [1.50, 2.40] | <0.001 |
| CBC-derived indicators | |  | | |  |
| NLR | 2.12 [1.60, 2.80] | | 2.00 [1.55, 2.59] | 2.31 [1.68, 3.10] | <0.001 |
| dNLR | 1.52 [1.18, 1.90] | | 1.45 [1.16, 1.83] | 1.59 [1.19, 2.00] | <0.001 |
| MLR | 0.27 [0.21, 0.36] | | 0.25 [0.20, 0.31] | 0.31 [0.24, 0.41] | <0.001 |
| NMLR | 2.40 [1.83, 3.15] | | 2.25 [1.77, 2.89] | 2.62 [1.96, 3.47] | <0.001 |
| SIRI, 10^3^/μL | 1.18 [0.80, 1.69] | | 1.03 [0.74, 1.49] | 1.33 [0.92, 1.92] | <0.001 |
| SII, 10^3^/μL | 546.75 [392.25, 764.06] | | 546.75 [396.22, 743.88] | 546.52 [386.05, 790.20] | 0.617 |

Abbreviations: PIR, poverty income ratio; NLR, neutrophil-to-lymphocyte ratio; dNLR, derived neutrophil-to-lymphocyte ratio; MLR, monocyte-to-lymphocyte ratio; NMLR, neutrophil-monocyte to lymphocyte ratio; SIRI, systemic inflammatory response index; SII, systemic immune-inflammation index; CBC, complete blood cell.

Continuous variables without a normal distribution are presented as medians [interquartile ranges]. Categorical variables are presented as numbers (percentages). The Benjamini-Hochberg method was used to adjust p values for multiple testing.

**Table S3.** HRs (95% CIs) of all-cause mortality according to quartiles of complete blood cell (CBC) counts among adults with sarcopenia in NHANES 1999–2006.

|  | Quartiles of CBC counts | | | |  |
| --- | --- | --- | --- | --- | --- |
|  | Quartile 1 | Quartile 2 | Quartile 3 | Quartile 4 | *P* _trend_ |
| WBC |  |  |  |  |  |
| Crude | 1 [Reference] | 0.959 (0.788-1.168) | 0.883 (0.729-1.070) | 0.949 (0.779-1.154) | 0.461 |
| Model 1 | 1 [Reference] | 0.976 (0.801-1.189) | 0.919 (0.758-1.116) | 1.130 (0.926-1.378) | 0.461 |
| Model 2 | 1 [Reference] | 0.942 (0.771-1.149) | 0.839 (0.688-1.023) | 0.996 (0.812-1.223) | 0.621 |
| NEU |  |  |  |  |  |
| Crude | 1 [Reference] | 1.134 (0.930-1.382) | 1.033 (0.843-1.264) | 1.234 (1.013-1.503) | 0.156 |
| Model 1 | 1 [Reference] | 1.134 (0.929-1.385) | 1.045 (0.851-1.282) | 1.335 (1.092-1.632) | 0.038 |
| Model 2 | 1 [Reference] | 1.092 (0.892-1.337) | 0.985 (0.800-1.213) | 1.186 (0.964-1.459) | 0.303 |
| MON |  |  |  |  |  |
| Crude | 1 [Reference] | 1.276 (1.063-1.533) | 1.285 (1.037-1.592) | 1.779 (1.473-2.148) | <0.001 |
| Model 1 | 1 [Reference] | 1.028 (0.853-1.238) | 1.018 (0.818-1.267) | 1.281 (1.053-1.558) | 0.062 |
| Model 2 | 1 [Reference] | 1.022 (0.847-1.233) | 0.944 (0.756-1.179) | 1.232 (1.010-1.502) | 0.171 |
| LYM |  |  |  |  |  |
| Crude | 1 [Reference] | 0.553 (0.458-0.669) | 0.542 (0.450-0.653) | 0.493 (0.403-0.604) | <0.001 |
| Model 1 | 1 [Reference] | 0.620 (0.513-0.750) | 0.725 (0.601-0.875) | 0.774 (0.631-0.949) | 0.027 |
| Model 2 | 1 [Reference] | 0.577 (0.476-0.699) | 0.676 (0.559-0.819) | 0.715 (0.581-0.881) | 0.004 |

The Benjamini-Hochberg method was used to adjust p values for multiple testing. Model 1 was adjusted as age (<39, 40-59, or >59), sex (male or female), and race/ethnicity (Mexican American, Other Hispanic, Non-Hispanic White, Non-Hispanic Black or Other); Model 2 was adjusted as model 1 plus education level (below high school, high school, or above high school), family poverty income ratio (≤1.0, 1.1–3.0, or >3.0), drinking status (nondrinker, low-to-moderate drinker, or heavy drinker), smoking status (never smoker, former smoker, or current smoker), physical activity (inactive, insufficiently active, or active), total energy intakes (in quartiles), self-reported diabetes (yes or no), and self-reported hypertension (yes or no).

**Table S4.** HRs (95% CIs) of cardiovascular mortality according to quartiles of complete blood cell (CBC) counts among adults with sarcopenia in NHANES 1999–2006.

|  | Quartiles of CBC counts | | | |  |
| --- | --- | --- | --- | --- | --- |
|  | Quartile 1 | Quartile 2 | Quartile 3 | Quartile 4 | *P* _trend_ |
| WBC |  |  |  |  |  |
| Crude | 1 [Reference] | 1.208 (0.816-1.788) | 0.944 (0.633-1.409) | 1.125 (0.755-1.675) | 0.914 |
| Model 1 | 1 [Reference] | 1.218 (0.821-1.806) | 0.963 (0.644-1.441) | 1.319 (0.882-1.972) | 0.576 |
| Model 2 | 1 [Reference] | 1.126 (0.756-1.677) | 0.827 (0.546-1.252) | 1.084 (0.717-1.640) | 0.914 |
| NEU |  |  |  |  |  |
| Crude | 1 [Reference] | 1.402 (0.938-2.095) | 1.148 (0.754-1.748) | 1.449 (0.966-2.172) | 0.332 |
| Model 1 | 1 [Reference] | 1.361 (0.907-2.043) | 1.111 (0.726-1.700) | 1.501 (0.994-2.267) | 0.319 |
| Model 2 | 1 [Reference] | 1.232 (0.817-1.859) | 0.982 (0.637-1.515) | 1.232 (0.806-1.885) | 0.718 |
| MON |  |  |  |  |  |
| Crude | 1 [Reference] | 1.262 (0.850-1.874) | 1.756 (1.164-2.650) | 2.681 (1.878-3.827) | <0.001 |
| Model 1 | 1 [Reference] | 0.981 (0.656-1.466) | 1.333 (0.875-2.030) | 1.810 (1.248-2.624) | 0.006 |
| Model 2 | 1 [Reference] | 0.950 (0.634-1.425) | 1.157 (0.754-1.777) | 1.694 (1.163-2.466) | 0.021 |
| LYM |  |  |  |  |  |
| Crude | 1 [Reference] | 0.619 (0.422-0.908) | 0.686 (0.477-0.986) | 0.501 (0.329-0.762) | 0.008 |
| Model 1 | 1 [Reference] | 0.700 (0.477-1.027) | 0.953 (0.660-1.375) | 0.822 (0.538-1.255) | 0.718 |
| Model 2 | 1 [Reference] | 0.660 (0.448-0.974) | 0.881 (0.606-1.282) | 0.764 (0.496-1.176) | 0.576 |

The Benjamini-Hochberg method was used to adjust p values for multiple testing. Model 1 was adjusted as age (<39, 40-59, or >59), sex (male or female), and race/ethnicity (Mexican American, Other Hispanic, Non-Hispanic White, Non-Hispanic Black or Other); Model 2 was adjusted as model 1 plus education level (below high school, high school, or above high school), family poverty income ratio (≤1.0, 1.1–3.0, or >3.0), drinking status (nondrinker, low-to-moderate drinker, or heavy drinker), smoking status (never smoker, former smoker, or current smoker), physical activity (inactive, insufficiently active, or active), total energy intakes (in quartiles), self-reported diabetes (yes or no), and self-reported hypertension (yes or no).

**Table S5.** HRs (95% CIs) of all-cause and cardiovascular mortality according to quartiles of CBC-derived inflammatory biomarkers levels among adults with sarcopenia after excluding participants who died within two years of follow-up in NHANES 1999–2006 (n=6262).

|  | Quartiles of CBC-derived inflammatory biomarkers levels | | | |  |
| --- | --- | --- | --- | --- | --- |
|  | Quartile 1 | Quartile 2 | Quartile 3 | Quartile 4 | *P* _trend_ |
| **All-cause mortality** | |  |  |  |  |
| NLR | 1 [Reference] | 0.826 (0.659-1.034) | 0.878 (0.704-1.096) | 1.227 (0.998-1.510) | 0.038 |
| dNLR | 1 [Reference] | 0.754 (0.603-0.943) | 0.901 (0.728-1.114) | 1.214 (0.989-1.490) | 0.036 |
| MLR | 1 [Reference] | 1.063 (0.834-1.355) | 1.166 (0.923-1.474) | 1.544 (1.221-1.952) | <0.001 |
| NMLR | 1 [Reference] | 0.857 (0.682-1.076) | 1.005 (0.806-1.253) | 1.266 (1.026-1.563) | 0.018 |
| SIRI | 1 [Reference] | 1.034 (0.816-1.310) | 1.335 (1.066-1.673) | 1.437 (1.148-1.799) | <0.001 |
| SII | 1 [Reference] | 0.880 (0.716-1.082) | 0.860 (0.696-1.063) | 0.978 (0.797-1.201) | 0.874 |
| **Cardiovascular mortality** | |  |  |  |  |
| NLR | 1 [Reference] | 1.078 (0.688-1.688) | 0.776 (0.483-1.245) | 1.203 (0.781-1.854) | 0.736 |
| dNLR | 1 [Reference] | 0.878 (0.568-1.358) | 0.797 (0.515-1.232) | 0.998 (0.650-1.533) | 0.906 |
| MLR | 1 [Reference] | 1.056 (0.624-1.789) | 1.228 (0.745-2.024) | 1.823 (1.117-2.975) | 0.018 |
| NMLR | 1 [Reference] | 1.051 (0.666-1.660) | 0.868 (0.543-1.387) | 1.252 (0.811-1.933) | 0.520 |
| SIRI | 1 [Reference] | 1.320 (0.808-2.156) | 1.143 (0.695-1.881) | 1.692 (1.059-2.704) | 0.069 |
| SII | 1 [Reference] | 0.760 (0.508-1.137) | 0.645 (0.420-0.989) | 0.679 (0.447-1.030) | 0.128 |

The Benjamini-Hochberg method was used to adjust p values for multiple testing. Model 1 was adjusted as age (<39, 40-59, or >59), sex (male or female), and race/ethnicity (Mexican American, Other Hispanic, Non-Hispanic White, Non-Hispanic Black or Other); Model 2 was adjusted as model 1 plus education level (below high school, high school, or above high school), family poverty income ratio (≤1.0, 1.1–3.0, or >3.0), drinking status (nondrinker, low-to-moderate drinker, or heavy drinker), smoking status (never smoker, former smoker, or current smoker), physical activity (inactive, insufficiently active, or active), total energy intakes (in quartiles), self-reported diabetes (yes or no), and self-reported hypertension (yes or no).

**Table S6.** HRs (95% CIs) of all-cause and cardiovascular mortality according to quartiles of CBC-derived inflammatory biomarkers levels among adults with sarcopenia after excluding participants who had cancer history at baseline in NHANES 1999–2006 (n=5702).

|  | Quartiles of CBC-derived inflammatory biomarkers levels | | | |  |
| --- | --- | --- | --- | --- | --- |
|  | Quartile 1 | Quartile 2 | Quartile 3 | Quartile 4 | *P* _trend_ |
| **All-cause mortality** | |  |  |  |  |
| NLR | 1 [Reference] | 0.840 (0.663-1.063) | 0.925 (0.733-1.167) | 1.223 (0.983-1.520) | 0.039 |
| dNLR | 1 [Reference] | 0.801 (0.634-1.013) | 0.943 (0.753-1.181) | 1.276 (1.029-1.583) | 0.019 |
| MLR | 1 [Reference] | 0.886 (0.682-1.153) | 1.114 (0.872-1.425) | 1.370 (1.066-1.762) | <0.001 |
| NMLR | 1 [Reference] | 0.891 (0.702-1.130) | 1.007 (0.798-1.272) | 1.282 (1.028-1.599) | 0.019 |
| SIRI | 1 [Reference] | 1.051 (0.821-1.347) | 1.315 (1.036-1.668) | 1.422 (1.122-1.802) | <0.001 |
| SII | 1 [Reference] | 0.871 (0.701-1.083) | 0.879 (0.703-1.098) | 1.033 (0.833-1.282) | 0.744 |
| **Cardiovascular mortality** | |  |  |  |  |
| NLR | 1 [Reference] | 1.099 (0.691-1.749) | 0.945 (0.589-1.518) | 1.276 (0.820-1.983) | 0.419 |
| dNLR | 1 [Reference] | 0.932 (0.595-1.459) | 0.913 (0.588-1.417) | 1.116 (0.725-1.715) | 0.680 |
| MLR | 1 [Reference] | 0.889 (0.513-1.541) | 1.098 (0.657-1.835) | 1.695 (1.023-2.809) | 0.019 |
| NMLR | 1 [Reference] | 1.099 (0.686-1.759) | 0.988 (0.615-1.589) | 1.359 (0.873-2.115) | 0.294 |
| SIRI | 1 [Reference] | 1.154 (0.694-1.918) | 1.168 (0.708-1.928) | 1.756 (1.095-2.814) | 0.024 |
| SII | 1 [Reference] | 0.845 (0.562-1.271) | 0.666 (0.428-1.035) | 0.846 (0.559-1.281) | 0.380 |

The Benjamini-Hochberg method was used to adjust p values for multiple testing. Model 1 was adjusted as age (<39, 40-59, or >59), sex (male or female), and race/ethnicity (Mexican American, Other Hispanic, Non-Hispanic White, Non-Hispanic Black or Other); Model 2 was adjusted as model 1 plus education level (below high school, high school, or above high school), family poverty income ratio (≤1.0, 1.1–3.0, or >3.0), drinking status (nondrinker, low-to-moderate drinker, or heavy drinker), smoking status (never smoker, former smoker, or current smoker), physical activity (inactive, insufficiently active, or active), total energy intakes (in quartiles), self-reported diabetes (yes or no), and self-reported hypertension (yes or no).

**Supplementary references**

S1. Cruz-Jentoft AJ, Sayer AA: Sarcopenia. Lancet (London, England) 2019, 393(10191):2636-2646. <https://doi.org/10.1016/S0140-6736(19)31138-9>

S2. Cruz-Jentoft AJ, Bahat G, Bauer J, Boirie Y, Bruyere O, Cederholm T, Cooper C, Landi F, Rolland Y, Sayer AA et al: Sarcopenia: revised European consensus on definition and diagnosis. Age Ageing 2019, 48(1):16-31. <https://doi.org/10.1093/ageing/afy169>

S3. Furman D, Campisi J, Verdin E, Carrera-Bastos P, Targ S, Franceschi C, Ferrucci L, Gilroy DW, Fasano A, Miller GW et al: Chronic inflammation in the etiology of disease across the life span. Nat Med 2019, 25(12):1822-1832. <https://doi.org/10.1038/s41591-019-0675-0>

S4. Suzuki K: Chronic Inflammation as an Immunological Abnormality and Effectiveness of Exercise. Biomolecules 2019, 9(6). <https://doi.org/10.3390/biom9060223>

S5. Gu W, Tian Z, Tian W, Song Y, Qi G, Qi J, Sun C: Association of rest-activity circadian rhythm with chronic respiratory diseases, a cross-section survey from NHANES 2011-2014. Respir Med 2023, 209:107147. <https://doi.org/10.1016/j.rmed.2023.107147>

S6. Fadeyev K, Nagao-Sato S, Reicks M: Nutrient and Food Group Intakes among U.S. Children (2-5 Years) Differ by Family Income to Poverty Ratio, NHANES 2011-2018. International journal of environmental research and public health 2021, 18(22). <https://doi.org/10.3390/ijerph182211938>

S7. Qiu Z, Chen X, Geng T, Wan Z, Lu Q, Li L, Zhu K, Zhang X, Liu Y, Lin X et al: Associations of Serum Carotenoids With Risk of Cardiovascular Mortality Among Individuals With Type 2 Diabetes: Results From NHANES. Diabetes Care 2022, 45(6):1453-1461. <https://doi.org/10.2337/dc21-2371>
