# Supplementary material for: Associations of CBC-Derived inflammatory indicators with sarcopenia and mortality in adults: evidence from Nhanes 1999 ∼ 2006
Source: BMC Geriatr. 2024 May 16;24:432. doi: 10.1186/s12877-024-05012-2 (PMC11100216; doi:10.1186/s12877-024-05012-2)
Supplement: Supplementary file 2 — Supplementary Material 2 [file 12877_2024_5012_MOESM2_ESM.docx]

**GPR TRANSFER - please note that we have received the offer of guaranteed peer-review from BMC Medicine, BMED-D-24-00762**

Dear Editor-in-Chief,

I am writing to submit our manuscript entitled ***"Associations of Complete Blood Cell Count (CBC)-Derived Inflammatory Indicators with Sarcopenia and Mortality in Adults:Evidence from Nhanes 1999~2006"*** for consideration for publication in the ***BMC Geriatrics***.

In this study, we aimed to investigate the links of complete blood cell count (CBC) parameters and CBC-derived inflammatory indicators with sarcopenia and mortality. Using data extracted from the 1999-2006 National Health and Nutrition Examination Survey (NHANES), and mortality events ascertained through the National Death Index up to December 31, 2019, we assessed various CBC-derived inflammatory indicators and their association with sarcopenia and mortality. Our analysis included the neutrophil-to-lymphocyte ratio (NLR), derived neutrophil-to-lymphocyte ratio (dNLR), monocyte-to-lymphocyte ratio (MLR), neutrophil-monocyte to lymphocyte ratio (NMLR), systemic inflammatory response index (SIRI), and systemic immune-inflammation index (SII).

The findings of our study revealed significant associations between elevated levels of these CBC-derived inflammatory indicators and both the prevalence of sarcopenia and the risk of all-cause and cardiovascular mortality in adults. Particularly, MLR emerged as the most robust predictor of all-cause and cardiovascular mortality in the population with sarcopenia.

We believe that our study's findings make a valuable contribution to the understanding of the role of inflammation in the development of sarcopenia and its implications for mortality in adults. We are confident that our manuscript aligns with the scope and interests of the ***BMC Geriatrics***, and we hope that our research will be considered for publication.

Thank you for considering our manuscript for publication, and we look forward to the opportunity to contribute to the journal's scientific discourse.

Best Regards.

Yours Sincerely,

Botang Guo, PhD & Dongsheng Hu, PhD

Department of General Practice, The Affiliated Luohu Hospital of Shenzhen University Medical School, Shenzhen, People’s Republic of China

Telephone: +86-755-86671951 Fax: +86-755-86671906

Email: dongshenghu563@126.com
